# Annotations

**Published:** 1889-08-17

**Authors:** 


### Annotations.

**7,000 made Happy.**

There are now more than 7,000 London children enjoying the delights of country cottage homes under the auspices of the Children's Country Holidays Fund. We cannot help wondering whether any member of the committee or any subscriber is half so happy as hundreds of these poor children are. Never before, we believe, have so many little ones been sent to play in the woods and fields, and to pluck the flowers that grow in such abundance by every roadside. There was a time when the population of London itself did not number seven thousand; and even now the capital town of several English and Scotch counties does not contain more than half that number. Seven thousand, indeed, is quite an army, large enough, if it consisted of true British warriors, to conquer the Isle of Wight or even the Calf of Man. Whilst the "happiness" view of the question will appeal most strongly to the majority of man and womankind, the medical instinct turns to the practical aspect of improved appetite, purified blood, strengthened body, and enlarged mind. The little Londoner, cooped up year in and year out in wretched streets and a more wretched home, becomes almost as limited in ideas as a London dog, or cat, or sparrow. The street is his world. Of the sense of space which the country boy experiences from his fields and hills, and of the still more infinite sense of space which he gains from daily looking at the rolling clouds and the measureless skies, the poor little cockney knows nothing. What a miserable caricature of God's universe is a London slum! And yet that is the universe of tens of thousands of London boys and girls. How can they be anything but narrow and stunted in mind or soul, just as they are narrow and stunted in chest and back? In sending so many as 7,000 out into the country at one time the Children's Country Holidays Fund are doing a great and noble work. What a pity it is they cannot send out seventy thousand instead of seven! They might if readers and other kind-hearted people would send them the money. The offices of the society are at 10, Buckingham-street, Strand, W.C.

**Palpitation.**

According to the *Medical Annual*, the treatment of palpitation should be "moral, hygienic, and medical." As the *Medical Annual* is professedly an organ for the propagation of new methods of treatment, this view may be considered the latest pronouncement of science on the subject of palpitation. The *Annual* not only gives the three lines of treatment named, but also says that the order of their importance is as indicated. The most important part of the treatment is therefore the "moral," and the least important the "medical," or, as it would be more properly expressed, the "medicinal," for "moral" treatment is "medical," in that it is recommended by the best medical science. Palpitation is undoubtedly a most distressing trouble, and in aged people may be dangerous as well as alarming. It is usually a disease of nervous origin, and is very much more common in women than men. The heart is supplied with nerve centres of its own, and these are of the nature of involuntary ganglia, or ganglia that cannot exercise will, as the brain can. It is true that nerves proceeding from the brain check and restrain the action of the heart's own nerve centres. But great mental emotion, especially mental excitement, is apt to increase very much the discharge of nerve energy from the heart's own ganglia, whilst the checking action of the pneumo-gastric nerves may remain as before, or even be diminished. In these circumstances the heart is like a runaway horse, whipped up with all possible fierceness by a strong man, whilst a much weaker man is holding the reins. The result is that the heart will continue to palpitate, or run away with tremendous speed until one of two things happens. Either the nerve ganglia will be exhausted, or the checking action will be increased; and then the palpitation will cease and a period of depression set in. This being the explanation, it is easy to see why the first and most important part of treatment should be "moral." The sufferer from palpitation should avoid excitement as she would a pestilence. Especially should she refrain from getting into a rage. Passionate people often endure agonies with palpitation, which is a just punishment for their irrationality. If they want to escape punishment they must learn to control their tempers. Of course, there are people, not passionate, whose nerves are naturally sensitive, and whose sensitiveness has been increased by grief, worry, and anxiety of various kinds. Sometimes a very little trouble, such as a worrying letter, a disagreement with a friend, and so on, is sufficient to give them palpitation. For these, hygiene and medicine may be necessary, but especially hygiene. They should take as much physical exercise as they are able to sustain; spend a great part of their time in the open air; live in cheerful society, and have frequent changes of scene and circumstance. They should particularly avoid food that excites indigestion and nervousness. Strong tea or coffee, or stimulants, are among their worst enemies. Drugs, except when ordered by the doctor, should not be indulged in. There is no doubt that a large proportion of persons who suffer from palpitation may, by the observance of these rules, keep their enemy thoroughly well in check.

**Awake at Last.**

The general practitioner is awake at last on the question of hospital abuse. Dr. R. Rentoul, of Liverpool, is to bring forward a series of resolutions at the forthcoming meeting of the British Medical Association dealing with the whole question. These resolutions are generally approved not only by a large number of family practitioners, but also by men of the first eminence in the profession, including Sir E. H. Sieveking, Dr. Matthews Duncan, Dr. R. Quain, Dr. Bridgwater, Dr. B. W. Richardson, Dr. Robert Barnes, Dr. W. T. Gairdner, Mr. Lawson Tait, Dr. Graily Hewitt, and many others. A still more striking evidence of the determination of the general practitioner to put an end to injurious hospital competition is furnished by the memorial of Mr. Hugh Woods, of Highgate, published in last week's *British Medical Journal*. That memorial sets forth in energetic language the grievances and the purposes of general practitioners. The remarkable circumstance about it, however, is not its strength of language, nor even its abstract justice, but the fact that it is signed by nearly every practitioner in London within a radius of two miles from Mr. Woods' own house. In other words, between fifty and sixty *bonâ fide* general practitioners, immediate neighbours of Mr. Woods, have expressed their entire concurrence in his steadfast determination to bring about a radical reform. In this mere note, we cannot give Mr. Woods' protest at length or offer any comments upon it. But that the movement is full of significance is unquestionable. We have always maintained the necessity for a root and branch reformation, at least of the out-patient department, and have as strongly insisted upon the fact that the business is really a doctor's question. So long as general practitioners were apathetic, hospital physicians and surgeons were apathetic; and so long as hospital physicians and surgeons were apathetic, members of committee were practically powerless, much as they might deplore the admitted abuses of the present system. Now that general practitioners are arousing themselves there is hope of better things. It is only a question of whether or not they will stand by each other in sufficient numbers, and with adequate determination. If they can unite and follow competent leadership, they will carry the day, and that without difficulty. If, on the other hand, they permit apathy and jealousy to rule them, they will find themselves deeper in the mire than they were before.

